# Effect of Microcurrent Stimulation on Pain, Shoulder Function, and Grip Strength in Early Post-Operative Phase after Rotator Cuff Repair

**DOI:** 10.3390/medicina57050491

**Published:** 2021-05-13

**Authors:** Donghyun Yi, Hwanyong Lim, Jongeun Yim

**Affiliations:** 1 Department of Physical Therapy, Graduate School of Sahmyook University, Seoul 01795, Korea; yidonghyun89@daum.net (D.Y.); goldlhy@hanmail.net (H.L.); 2 Department of Physical Therapy, Sahmyook University, Seoul 01795, Korea

**Keywords:** rotator cuff repair, microcurrent stimulation, range of motion, grip strength, shoulder pain

## Abstract

*Background and Objectives*: The purpose of this study was to investigate the effects of microcurrent stimulation on pain, shoulder function, and grip strength in patients with rotator cuff repair. *Materials and Methods:* This randomized single-blind controlled trial was conducted on inpatients of the rehabilitation department, and included 28 patients who underwent rotator cuff repair. Participants were randomly assigned to the experimental group (n = 14), treated with microcurrent stimulation, and the control group (n = 14), treated with false microcurrent stimulation. The microcurrent stimulation administered to the experimental group underwent general physical therapy and microcurrent stimulation three times a week for 4 weeks. *Results*: Changes in pain, range of motion in shoulder, simple shoulder test, and grip strength were assessed before and after the intervention. Both groups showed a significant decrease in pain and shoulder function (t = 27.412, 22.079, 19.079, and 18.561; *p* < 0.001), and grip strength showed a significant increase (t = −8.251 and −9.946; *p* < 0.001). The experimental group that underwent microcurrent stimulation exhibited a significant effect on pain, shoulder function, and grip strength compared with the control group that underwent false microcurrent stimulation (t = −2.17, −2.22, and 2.213; *p* = 0.039, 0.035, and 0.036). *Conclusions:* This study confirmed that microcurrent stimulation is effective for the treatment of rotator cuff repair patients.

## 1. Introduction

Rotator cuff tears frequently occur because of excessive exercise, labor, and excessive work in daily life [1]. Pathological changes in a tendon result from vascular and metabolic disorders, as well as from changes in collagen fibers, caused by a degenerative rupture [2]. The structure of the tendon is closely related to the function and minimization of the risk of injuries and failures. Tendons are characterized by a high mechanical strength and rigid structure with a fibrous elasticity. The tendon is composed of the extracellular matrix and various types of cells, but is mainly composed of tendon cells, which are responsible for the synthesis of collagen molecules and for the balance of the extracellular matrix. The extracellular matrix is responsible for the mechanical functions of tendons [3]. Rupture of the rotator cuff may limit the functional movement of the upper extremities because of extreme pain and deterioration of the shoulder stability [4].

Rotator cuff tears can be treated by surgical and non-surgical methods, and non-surgical treatment includes anti-inflammatory medications, postural changes, strength and stretching exercises, and conservative physical therapy for pain relief [5]. Surgical methods are considered when non-surgical methods are not effective against reducing the symptoms [6]. The cases of treatment for rotator cuff tears are gradually increasing. As arthroscopy was only developed recently, surgical methods for rotator cuff repair have been performed frequently. Therefore, a method is necessary in order to overcome postoperative pain and rehabilitation [7]. Previous studies on the treatment of patients with rotator cuff repairs have provided promising clinical outcomes with personalized postoperative rehabilitation protocol [8,9].

Many patients undergoing surgical treatment may not be able to move the surgical site because of fear of postoperative pain and anxiety regarding re-injury, resulting in stiffness of the shoulder joint [10] that further leads to a reduction in muscular blood circulation, removal of waste, flow of nutrients, and causes pain and muscle fatigue. Thus, surgical methods require a lot of postoperative recovery time [11]. Even if exercise intervention is effective at alleviating pain and improving motor function, it is difficult to apply immediately after surgery. Although active exercise intervention is necessary for a patient in the initial time period after surgery [12], exercise intervention is difficult at the initial stage because of joint fixation of the surgical area, and this requires electrotherapy with electrical stimulation through the manipulation of various variables [13]. Microcurrent stimulation has the advantage of rarely causing muscle contractions and uncomfortable sensations to the skin, and has only a few electrical side effects; hence, this stimulation is suitable for patients who have limited mobility after surgery [14]. The stimulation is also referred to as bioelectric stimulation, because it cannot sense the flow of current that is used as a non-sensitized stimulus or a low current at a bioelectric level. The basis of the stimulation is the ion channel and cellular communication that stimulate cell activity by normalizing the cell’s electrical environment [15]. Several clinical studies on this stimulation have shown that it gradually increases the intracellular protein synthesis rate, adenosine triphosphate re-synthesis, and DNA replication. Furthermore, it activates the differentiation, proliferation, and migration of cells by facilitating the tissue healing and recovery process [16].

Shah and Farrow (2013) reported that the healing rate of soft tissue treated with microcurrent stimulation was faster than that of shortwave diathermy when these two treatments were applied to different groups experiencing soft tissue damage of the ankle [17]. The application of microcurrent stimulation on the wound areas of patients with trauma not only shortened the suture time and hospitalization, but also reduced pain [18].

Therefore, the purpose of this study was to investigate the effects of microcurrent stimulation on pain, shoulder function, and grip force in rotator cuff repair patients.

## 2. Materials and Methods

### 2.1. Participants

This study included 28 patients who were admitted to K hospital in Seoul and underwent rotator cuff repair. The number of participants was calculated using G-Power, with a power (0.80) and effect size (0.50) based on a previous paper [19]. The criteria for the selection of patients were 20–70 years of age and 1–2 weeks following arthroscopic surgery for a diagnosis of degenerative rupture of the supraspinatus tendon. The patients had no problems in communicating with the physical therapist and no postoperative symptoms of inflammation or side effects. The criteria for exclusion were patients with cardiac diseases, contraindicated for electrotherapy, artificial pacemaker, seizures, problems with communication due to mental problems or lack of understanding, and pregnancy. After providing a detailed explanation about the study, we obtained written consent forms from 28 healthy participants. The demographic data of the participants are shown in Table 1.

### 2.2. Intervention

This study was conducted over four weeks. Pre- and post-test evaluations for the patients’ pain, shoulder function, and grip were conducted. Among the 40 patients with rotator cuff repair voluntarily participating in this study, 28 participants met the selection criteria, and were randomly divided into the experimental group and control group (n = 14, each) using drawing lots to minimize selection bias. The experimental group underwent general physical therapy and microcurrent stimulation three times a week for four weeks. The control group received general physical therapy and sham microcurrent stimulation three times a week for four weeks. This study was conducted with the approval of the Research Ethics Committee of Sahmyook University.

#### 2.2.1. Microcurrent Stimulation

The microcurrent stimulation was performed with the MCPLUS-17062215 (KOR, 2017) with a 2.8 cm electrode at a fixed frequency of 30 pps, pulsation frequency of 5 pps, and intensity of 25 Hz for 15 min. In the experimental group, two pads were attached near the surgical site as close as possible [19].

#### 2.2.2. Sham Microcurrent Stimulation

In the control group, two pads were attached close to the surgical site, the same as for the experimental group, but false microcurrent stimulation was performed, wherein no actual current flowed.

#### 2.2.3. General Physical Therapy

According to the prescription, general physical therapy was performed in the experimental and control groups using cooling therapy (15 min) and manual exercise therapy (15 min) within the range of joint movement without pain.

### 2.3. Outcome Measurement

#### 2.3.1. Pain

The visual analogue scale (VAS) was used to assess the subjective pain levels in the patients. It allows patient’s subjective pain to be displayed on a 10-cm long line that is measured with a ruler. The amount of pain is objectified and quantified [20]. The VAS reportedly has a very high level of intra-measurement reliability (r = 1.00) and inter-measurement reliability (r = 0.99) [21].

#### 2.3.2. Grip Strength

The grip strength of the participants was measured using a Gripmeter (Jamar^®^ hydraulic hand dynamometer, Model 5030J1, Chicago, IL, USA, 2003). The grip strength was assessed by keeping the forearm of the participant in the 90-degree upright position and applying maximum force. Prior training for the measurement operation was conducted. After repeating the measurement twice, the average value was used [22].

#### 2.3.3. Range of Motion

A smartphone digital inclinometer (Clinometer, version 3.7, Stephanskirchen, Germany, 2009) was used to assess the range of motion of the participants. Shoulder flexion and abduction range of motion were measured when the patient was active in the upright posture in the range of no pain. Because of the structural problems of unstable shoulders after surgery, we measured only flexion and abduction. Secondary injury such as re-injury could occur if internal rotation or external rotation were performed. Prior training for the measurement operation was conducted. After performing the measurement thrice, the average value was used. Regardless of diagnosis or surgery, all of the results were in agreement with the values obtained with the universal goniometer. Therefore, the smartphone digital inclinometer application can be used as a reliable shoulder range of motion tool [23].

#### 2.3.4. Simple Shoulder Test

A simple shoulder test (SST) was used to evaluate the shoulder joint function of the participants. SST is the most widely used shoulder questionnaire, developed by Washington University Hospital in the United States. It consists of 12 questions, and a 12-point score indicates that all items are considered inoperable. The lower the score, the better the shoulder joint function. The evaluator marked “yes” (0) or “no: (1) for the 12 questions. The SST has high inter-rater reliability (r = 0.97) and intra-rater reliability (r = 0.85) [24].

### 2.4. Data Analysis

Data were statistically analyzed with the Statistical Software for the Social Sciences (Windows SPSS/PC Statistics 23.0 software; SPSS Inc., Chicago, IL, USA). The participants’ general characteristics were analyzed using a chi-square test. The Shapiro–Wilk test was used to verify the normality of the data. The changes in pain, shoulder function, and grip strength after intervention in the two groups were compared using the paired *t*-test. Differences between the two groups were analyzed with the independent *t*-test. Statistical significance was set at α = 0.05.

## 3. Results

### 3.1. Pain

The changes in pain scores in the experimental group and control group before and after treatment of 4 weeks are shown in Table 2. The VAS scores significantly decreased in both the experimental and control groups after four weeks of treatment (*p* < 0.05). With respect to the VAS scores before and after treatment, the experimental group showed a statistically significant difference compared with the control group (*p* < 0.05).

### 3.2. Grip Strength

The changes in grip strength in the experimental group and control group before and after treatment for four weeks are shown in Table 2. Changes in grip strength were significant in both the experimental and control groups after four weeks of treatment (*p* < 0.05). With respect to the changes before and after treatment, the experimental group showed a statistically significant difference compared with the control group (*p* < 0.05).

### 3.3. Range of Motion

The results indicating the changes in the range of motion before and after treatment are shown in Table 3, and a significant difference was observed in the range of motion before and after treatment in both groups (*p* < 0.05). Furthermore, the experimental group showed a statistically significant difference compared with the control group (*p* < 0.05).

### 3.4. Simple Shoulder Test

The changes in shoulder function scores and the range of motion in the experimental group and control group before and after treatment for four weeks are shown in Table 3. A significant change was observed in the SST before and after treatment in both groups (*p* < 0.05), and the experimental group showed a statistically significant difference compared with the control group (*p* < 0.05).

## 4. Discussion

The rotator cuff is an important muscle group that is involved in the stability of the shoulder joint during movements, and its rupture may affect the functional movement of the upper extremities, resulting in shoulder instability and extreme pain [4]. It is difficult to perform exercise interventions to repair the rotator cuff because of the initial joint fixation after surgery. However, electrotherapy can be used safely, without joint motion, in the early phase after surgery [13]. Microcurrent stimulation causes less irritation to the surgical area, without any side effects, and is suitable for patients with limited movements because of the postoperative brace [14]. Therefore, this study aimed to investigate the influence of microcurrent stimulation in patients who underwent rotator cuff repair in the early phase after surgery and to verify its efficiency.

Twenty-eight rotator cuff repair patients who met the selection criteria were divided into two groups, and treatment was conducted for three days a week over four weeks. There was a significant improvement in pain, range of motion of shoulder, SST, and grip strength in both the experimental and control groups (*p* < 0.05).

Based on this study, the pain significantly reduced in the experimental group compared with the control group after four weeks of treatment with microcurrent stimulation (*p* < 0.05). Cho and Kim (2012) reported that 15 patients treated with a microcurrent of 25 mm intensity, a pulsation frequency of 5 pps, and frequency of 30 pps after total knee arthroplasty showed a significant difference in the pain between the non-treated and the treated groups (*p* < 0.05) [19]. Chung and Cho (2015) also found that microcurrent stimulation was very effective at significantly relieving pain in patients with degenerative knee arthritis [25]. Wahaj and Hafeez (2017) conducted clinical studies that showed that microcurrent stimulation was highly effective at alleviating the pain of the temporomandibular joint [26]. Maul and Borchard (2019) reported that microcurrent stimulation in 72 patients with facial pain due to sinus disease showed a reduction in their mean VAS score from 5.63 cm before treatment to 3.97 cm after treatment [27]. These studies showed consistent results regarding pain relief with microcurrent stimulation. Microcurrent stimulation can promote tissue healing, as new capillaries are formed and blood flow increases [28]. In Lee’s study [29], it was found that a substance produced by pain was removed by the local blood flow stimulated by the stimulation. Therefore, pain relief occurs when the rate of tissue healing increases because of the blood flow, causing removal of the substances causing pain (substance P).

The present study clearly shows the range of motion of the joints increased significantly in the experimental and control groups after four weeks of treatment (*p* < 0.05). Cho and Song (2014) compared the effects of cutaneous electrical and microcurrent stimulation on percutaneous nerves with delayed myalgia in 27 healthy participants using a 60 μA intensity and a pulsation frequency of 3 pps for 15 min. A comparison between the microcurrent group before and after treatment according to time showed a significant difference in the range of motion of the joint at flexion 48 h later [30]. Cho and Kim (2012) showed that 15 patients with total knee arthroplasty treated with microcurrent stimulation for 15 min showed significant therapeutic effects for the range of motion of the joints [19]. Casini and Selvi (2017) reported that microcurrent stimulation increases the likelihood of joint movement, as it increases gelatinous synthesis and promotes the healing of tissues, such as tendons, ligaments, and skin, affected by trauma [18]. Additionally, microcurrent stimulation can improve the general condition of a patient, such as movement, and shorten the tissue suturing time.

Thus, the increased range of motion is associated with increased gelatin synthesis, which promotes tissue healing and affects ligament and tendon healing. This shows that microcurrent stimulation had a significant effect on increased range of motion. 

It was found that SST showed a statistically significant reduction in the experimental group compared with the control group after four weeks of treatment (*p* < 0.05). In previous studies on the function of microcurrent stimulation, there was a significant difference in the movement of the elbow joint muscle function as a result of microcurrent stimulation in 15 patients with acute lateralepicondylitis (*p* < 0.01) [31]. Schmidt-Malan and Brinkman (2019) reported that microcurrent stimulation stimulates receptor proteins by opening Na+ and Ca^2^⁺ pathways in the cell membranes to stimulate the proliferation of cells such as chondrocytes, bone cells, fibroblasts, and vascular endothelial cells. The differentiation and migration of the cell process enhances functional improvement.

This study suggested that the improved function of the shoulder joint was due to the smooth supply of protein through active cell proliferation and migration. Therefore, the tissue healing was good and significantly affected the function.

According to this study, the grip strength significantly increased with the application of microcurrent stimulation in the experimental group compared with the control group after four weeks of treatment (*p* < 0.05).

The study by Jeon et al. [32] showed that microcurrent stimulation applied early in injury related to trauma-induced muscle damage was effective at reducing muscle tissue damage. In the study by Shah and Farrow [17], the rate of healing was higher after microcurrent stimulation in soft tissue injuries, such as in the ankles and muscles. Nessler and Mass (1987) found that applying a 7 μA microcurrent to the excised and cultured tendons of rabbits increased hydroxyproline by 255% and proline by 91%, which indicates that microcurrent was effective at promoting tendon healing [33].

Lambert and Marcus (2002) stated that the mechanism of action of microcurrent stimulation in the human body is closely related to the regulation of intercellular Ca^2^⁺ homeostasis by supplying electrical energy at the cellular level in injury-related diseases, and it is possible to promote the wound healing of tissues and to strengthen the muscles [34]. In the present study, the reasons for the increased grip strength were intracellular homeostasis, increased blood flow, and promoted metabolic progression, which caused rapid healing in the muscles, tendon junctions, and soft tissues. Hence, it is believed that microcurrent stimulation influenced the increase in grip strength as a result of pain reduction and shoulder stabilization.

The limitation of this study was that the total of four weeks for the experimental period for the application of microcurrent stimulation might not be enough to see the full effects of microcurrent stimulation. Moreover, it is impossible to control the drug use of the participants and it is difficult to generalize the results because of the small sample size. Additional research is needed, because personal characteristics such as age, gender, and smoking status, may have an impact on the outcomes. In addition, the type of surgery and the age range of the subject could affect the outcome. Therefore, in future studies, it will be necessary to distinguish and consider these variables.

## 5. Conclusions

Based on the results of this study, we can conclude that microcurrent stimulation has a positive an effect on pain, shoulder function, and grip strength in patients with rotator cuff repair. Therefore, microcurrent stimulation could be proposed as an effective treatment.

## Figures and Tables

**Table 1:** Physical characteristics of the subjects (*n* = 28).

	Experimental Group (*n* = 14)	Control Group (*n* = 14)
Age	54.64 ± 12.89	53.14 ± 9.94
Gender (male/female)	7/7	5/9
Height (cm)	165.50 ± 9.10	161.86 ± 9.35
Weight (kg)	63.36 ± 0.70	58.71 ± 10.29

Mean ± SD.

**Table 2:** Changes in pain and grip strength before and after treatment (*n* = 28).

		Experimental Group (*n* = 14)	Control Group (*n* = 14)	*t (p)*
	Pre	7.21 ± 0.70	7.50 ± 0.76	
VAS	Post	2.35 ± 0.63	3.21 ± 0.70	
	Difference	−4.86 ± 0.66	−4.29 ± 0.73	−2.17 (0.039)
	*t(p)*	27.41 (*p* < 0.001)	22.08 (*p* < 0.001)	
	Pre	20.50 ± 0.70	17.64 ± 8.86	
Grip	Post	23.93 ± 9.04	20.00 ± 8.34	
	Difference	3.43 ± 1.55	2.36 ± 0.93	2.21 (0.036)
	*t(p)*	−8.25 (*p* < 0.001)	−9.95 (*p* < 0.001)	

Mean ± SD; AS—visual analogue scale.

**Table 3:** Changes in shoulder function before and after treatment (*n* = 28).

		Experimental Group (*n* = 14)	Control Group (*n* = 14)	*t (p)*
	Pre	38.57 ± 8.73	32.93 ± 8.25	
ROM Flexion	Post	121.36 ± 6.74	112.00 ± 5.91	
	Difference	82.76 ± 7.39	76.07 ± 8.21	2.27 (0.031)
	*t(p)*	−41.90 (*p* < 0.001)	−34.66 (*p* < 0.001)	
	Pre	32.56 ± 7.72	30.29 ± 4.71	
ROM Abduction	Post	118.36 ± 8.90	108.29 ± 7.22	
	Difference	86.00 ± 8.38	78.00 ± 5.43	3.00 (0.006)
	*t(p)*	−38.38 (*p* < 0.001)	−53.70 (*p* < 0.001)	
	Pre	10.29 ± 0.83	10.79 ± 0.80	
SST	Post	6.29 ± 0.83	7.43 ± 1.09	
	Difference	−4.00 ± 0.78	−3.36 ± 0.74	−2.22 (0.035)
	*t(p)*	19.08 (*p* < 0.001)	18.56 (*p* < 0.001)	

Mean ± SD; ROM—range of motion; SST—simple shoulder test.
